# Fine-Grained Breast Cancer Classification With Bilinear Convolutional Neural Networks (BCNNs)

**DOI:** 10.3389/fgene.2020.547327

**Published:** 2020-09-04

**Authors:** Weihuang Liu, Mario Juhas, Yang Zhang

**Affiliations:** ^1^College of Science, Harbin Institute of Technology, Shenzhen, China; ^2^Faculty of Science and Medicine, University of Fribourg, Fribourg, Switzerland

**Keywords:** breast cancer, classification, histopathological images, convolutional neural networks, bilinear convolutional neural networks

## Abstract

Classification of histopathological images of cancer is challenging even for well-trained professionals, due to the fine-grained variability of the disease. Deep Convolutional Neural Networks (CNNs) showed great potential for classification of a number of the highly variable fine-grained objects. In this study, we introduce a Bilinear Convolutional Neural Networks (BCNNs) based deep learning method for fine-grained classification of breast cancer histopathological images. We evaluated our model by comparison with several deep learning algorithms for fine-grained classification. We used bilinear pooling to aggregate a large number of orderless features without taking into consideration the disease location. The experimental results on BreaKHis, a publicly available breast cancer dataset, showed that our method is highly accurate with 99.24% and 95.95% accuracy in binary and in fine-grained classification, respectively.

## Introduction

Breast cancer, the most often diagnosed cancer type and the leading cause of cancer-related death among women, was involved in more than 600,000 deaths and 2,000,000 new hospitalizations in 2018 Bray et al. (2018). Currently, analysis of histopathological images of breast cancer is still among the main methods of diagnosis. However, this method suffers from various shortcomings: (1) analysis by inexperienced doctors can lead to wrong diagnosis, (2) overwork may also lead to misdiagnosis, and (3) manual diagnosis is both time-consuming and laborious. Novel approaches for automatic diagnosis of the breast cancer with high accuracy and efficiency are therefore urgently needed. With the development of computer vision, automatic cancer image diagnosis has attracted a lot of attention from the scientific community. Moreover, the development of the slide scanning technology and collection of numerous digital histopathological images enabled computer-based analysis. Previous methods employed hand-crafted features to find a series of hyperplanes in the feature space that formed the optimal decision boundary for the high-dimensional feature space. Doyle et al. (2008) calculated more than 3,400 textural and structural features from breast tissue images, then used graph embedding for dimensionality reduction, and finally employed a support vector machine for cancer images identification. Kowal et al. (2013) extracted 42 morphological, topological and texture features from the segmented breast nucleus, and tested four different clustering methods, namely K-means, fuzzy C-means, competitive learning neural network and gaussian mixture model. Filipczuk et al. (2013) used k-nearest neighbor, naive bayes, decision tree, and support vector machine approaches to classify breast cancer based on segmented nucleus. Zhang Y. et al. (2014) proposed a nuclear principal component analysis based on manual features to classify benign and malignant breast cancer histopathology images. Wang P. et al. (2016) combined wavelet decomposition with multi-scale regional growth to obtain the region of interest. In this study, cells were segmented using a double strategy splitting model leading to extraction of four shape-based and 138 color-based features and breast cancer classification was achieved with support vector machine method.

Recently, deep learning has attracted attention in molecular (Le et al., 2019a,b; Do et al., 2020) and biomedical image analysis (Wang J. et al., 2016; Jiang et al., 2020; Li et al., 2020). In biomedical image analysis, CNNs represents the mainstream approach. Wang J. et al. (2016) developed a stacked denoising autoencoder to diagnose early breast cancer. Abdel-Zaher and Eldeib (2016) proposed an automated breast cancer diagnosis system based on deep belief networks. Cruz-Roa et al. (2017) proposed a simple CNNs- based method for detection of invasive breast cancer. Chougrad et al. (2018) used transfer learning instead of random initialization and fine-tuned VGG16 (Simonyan and Zisserman, 2014), ResNet (He et al., 2016) and InceptionV3 (Szegedy et al., 2016) for breast cancer detection. Khan et al. (2019) used GoogleNet (Szegedy et al., 2015), VGG16 and ResNet for extraction of low-level features and combined them for breast cancer classification. Campanella et al. (2019) used multiple instance learning-based deep learning approach to classify basal cell carcinoma, prostate and breast cancer, thus avoiding expensive and time-consuming pixel-wise manual annotations. Test results using 44,732 whole slide images from 15,187 patients resulted in areas under the curve above 0.98 for all cancer types. Babak et al. (2018) developed a cascaded CNNs to identify and distinguish tumor-associated stromal alterations from stroma associated with benign breast disease and assess stromal characteristics in varying grades of ductal carcinoma. Shen et al. (2019) developed a CNN method for breast cancer detection, where lesion annotations are required only in the pretrain stage, while the subsequent stages require only image-level labels. This eliminated the reliance of the method on rarely available lesion annotations. Joseph et al. (2019) described a proliferation tumor marker network, which can accurately detect the tumor area in immunohistochemistry (IHC)-stained breast cancer samples and identify regions of high proliferation using an activation filter map.

However, these earlier approaches suffer from various shortcomings. Firstly, the current classification of pathological images is mainly binary. This simplified classification is not sufficient for clinical diagnosis where different stages and disease types have to be identified. Secondly, contrary to macroscopic images where the target is located in a particular region, the lesions in histopathological images are widespread. Finally, the resolution of histopathological images is too large for direct analysis. By downsampling the image, CNNs learn only the overall image pattern, but not the patch differences. This can be solved by using a sliding window for selection of small patches for individual prediction and their subsequent combination for analysis, by a so called patch-based method. However, this method is time consuming and not suitable to establish a robust association between the patch-level and the image-level labels. Notably, the sliding window’s size and the patch must be carefully optimized as those will affect the model performance.

Fine-grained classification can be applied to distinguish between the small inter-class and large intra-class variances in histopathological images. Fine-grained image classification differentiates between hard-to-distinguish or similar subclasses in plants (Nilsback and Zisserman, 2008), animals (Catherine et al., 2011), and models of vehicles (Krause et al., 2013). Some approaches of histopathological image classification do not address the peculiarity of histopathological images and do not use specialized fine-grained classification methods (Han et al., 2017; Bardou et al., 2018; Gandomkar et al., 2018; Li et al., 2018; Yan et al., 2019). Previously, fine-grained image classification of histopathological images was shown to perform better than ordinary CNNs. Wang et al. (2017) used BCNNs for colorectal cancer histopathological image classification. In this study, histopathological images were first decomposed into hematoxylin and eosin stain components, and BCNNs were performed on the decomposed images. This method performed better than directly training the histopathological images with ordinary CNNs. Feature output variations were relatively large between different subclasses and small within the same subclass. The outputs from this study were embedded into the feature extraction process by Li et al. (2018). There are three specialized methods for fine-grained classification, namely fine-grained feature learning-based methods (Lin et al., 2015), object part annotation-based methods (Zhang N. et al., 2014), and visual attention-based methods (Jianlong et al., 2017). Object part annotation-based methods require hard-to-obtain additional annotations of the regions of interests. Consequently, we discuss the effectiveness of the other two methods, which require only the histopathological images’ label for classification.

**FIGURE 1:**
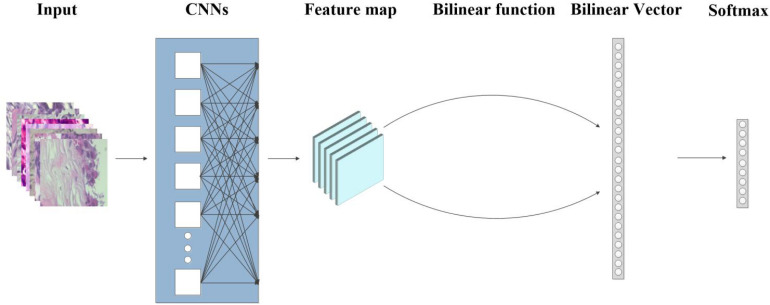
Flow graph of the proposed method of Bilinear Convolutional Neural Networks (BCNNs). The input image is first fed into the CNNs to get the feature maps, then the bilinear vector is obtained from the bilinear function, and finally flowed into the softmax layer for classification.

Here, we introduce BCNNs approach (Figure 1) for fine-grained breast cancer classification with improved interpretability and speed. This network uses bilinear pooling to obtain a large number of orderless features for strong translation invariant learning of the model, thus mitigating the uncertainty arising from the widespread location of the disease. In the experiments based on BreaKHis, we achieved the best performance in both fine-grained and binary classification. Furthermore, results in magnification-independent experiments suggests that our method is flexible with a strong scale invariant. Thus, different models do not have to be trained separately with different magnification factors. Experiments on in-house dataset indicate that our algorithm can be applied generally for breast cancer classification. Our fine-grained classification method provides more detailed information of histopathological images and can therefore assist doctors in the early diagnosis and treatment of the breast cancer.

## Materials and Methods

### Implementation

To verify the effectiveness of our model, we tested our model on BreaKHis, a publicly available dataset of breast cancer histopathological images. The dataset is available on https://web.inf.ufpr.br/vri/databases/breast-cancer-histopathological-database-breakhis. BreaKHis is a large-scale dataset that includes 7909 histopathological images taken from 82 patients, which is divided into two main classes, benign and malignant, and each is further divided into four different subclasses. The four benign tumors types are: adenosis, fibroadenoma, phyllodes tumor, and tubular adenoma. The four malignant tumors types are: ductal carcinoma, lobular carcinoma, mucinous carcinoma, and papillary carcinoma. Images are acquired with a resolution of 700 × 460 using different magnification factors of 40×, 100×, 200×, and 400× (Table 1). Besides data augmentation, we also utilize over-sampling to avoid overfitting and data imbalance problems.

**TABLE 1:** The number of images per class in BreaKHis.

		Magnification factor				
Class	Subclass	40×	100×	200×	400×	Total
Benign	Adenosis	114	113	111	106	444
	Fibroadenoma	253	260	264	237	1014
	Phyllodes tumor	149	150	140	130	569
	Tubular adenoma	109	121	108	115	453
Malignant	Ductal carcinoma	864	903	896	788	3451
	Lobular carcinoma	156	170	163	137	626
	Mucinous carcinoma	205	222	196	169	792
	Papillary carcinoma	145	142	135	138	560
Total		1995	2081	2013	1820	7909

We trained our model using the Keras framework with a Tesla K40C in an Ubuntu 16.04 system. Instead of randomly initializing our model, we transferred the weights trained on the ImageNet dataset and fine-tuned on our dataset. With pretraining, we could benefit from additional training data when domain-specific data was scarce, which is beneficial for many computer vision tasks. We use 5-fold cross-validation to evaluate the performance of the models. With this method, we randomly divide the data into five equal folds and the model is trained and tested 5 times to get the average accuracy, where each time it is trained on 4 folds and tested on the remaining fold. All histopathological images were resized to 224 × 224 before input into the network and data was augmented by random horizontal flipping, vertical flipping, height shifting, width sifting, translating, and rotating of the images. The hyperparameters are chosen using grid search, including learning rate, batch size and weight decay. We used mini-batch stochastic gradient descent as the optimizer with an initial learning rate of 0.1 and batch size of 16 to train our model. When the training loss did not decrease in 10 epochs, the learning rate was reduced by a factor of 10. Accuracy indicates the percentage of correctly classified samples among all samples and frames per second (FPS) represents the number of frames processed by the model per second. The codes that support the findings of this study are available on https://github.com/NiFangBaAGe/FBCNN.

### Backbone Networks

CNNs, which circumvent the complicated preprocessing of the image, have great capability to extract features with their special structure of local connection and weight sharing by inputting the original image directly. CNNs have unique advantages in image processing. They consist of neurons with trainable weights and bias constants, and have a special structure, which is called a convolutional layer. CNNs contain stacked convolutional layers that perform spatial operations on the image. Assuming that the size of input image *X* is *I*^∗^*J*, the number of convolution kernels in the convolutional layer is *H*, and the output and input channels are *L* and *K*, respectively, then the two-dimensional feature map’s convolution formula of the *l*th output channel is expressed as follows:

(1) $$c\,o\,n\,v\,{\left(X\right)}_{l}=\sum_{k=1}^{K}\sum_{i=1}^{i}\sum_{j=1}^{j}X^{k}\,\left(i,j\right)\,H^{k\,l}\,\left(i,j\right)$$

There are many well-known CNNs in image classification, including ResNet50, InceptionV3, InceptionResNetV2, etc. Increasing the number of layers does not improve classification performance when the CNNs reaches a certain depth. On the contrary, it will result in slower network convergence and less accurate classification. ResNet is designed to overcome this problem. ResNet uses a number of layers called residual block to learn the mapping of residuals between input and output, rather than using layers to directly learn the mapping between input and output like other CNNs. Evolved by InceptionV1 and InceptionV2 (Ioffe and Szegedy, 2015), one of the most important improvements of InceptionV3 is factorization, which factorizes a 7 × 7 convolution kernel into two convolutional kernels of size 1 × 7 and 7 × 1, and does the same operation for a 3 × 3 convolutional kernel, which speeds up the calculation. Furthermore, factorizing 1 convolutional layer into 2 convolutional layers increases the network depth and nonlinearity. InceptionResNetV2 combines the advantages of ResNet and InceptionV3, but the complexity is greatly increased. They all achieve excellent results in ImageNet competition and are widely used in various computer vision tasks to extract image features. We use ResNet50, InceptionV3, InceptionResNetV2 as backbone networks for feature-extracting to build our models.

### SE Block

SE Block was proposed by Hu et al. (2018). The network learns the weight according to the loss, which causes the effective feature maps’ weight to increase, and the ineffective or small effect feature maps’ weight to decrease, so that the model achieves better results. SE Block consists of two parts, Squeeze and Excitation. The Squeeze part uses the global pooling to integrate the input feature map of size *C*×*H*×*W* into the feature descriptor of size *C*×1×1 as in:

(2) $$s_{c}=F_{\text{sq}}\,\left(u_{c}\right)=\frac{1}{H\times W}\,\sum_{i=1}^{H}\sum_{j=1}^{W}u_{c}\,\left(i,j\right)$$

The Excitation part contains two fully connected layers using the sigmoid activation function. The fully connected layer fuses all the input feature information, and the sigmoid function maps the input to 0–1, which can be represented as:

(3) $$e=F_{\text{ex}}\,\left(s,W\right)=\sigma \,\left(g\,\left(s,W\right)\right)=\sigma \,\left(W_{2}\,\delta \,\left(W_{1}\,s\right)\right)$$

Where *s* is the global descriptor obtained by the Squeeze part, δ is the relu function, and *W*_*1*_ and *W*_*2*_ are the two fully connected layers. Finally, the weights of the individual channels of the input feature map E obtained are merged with the original features:

(4) $$F^{\prime }=F\,\left(u_{c},S_{c}\right)=S_{c}\times u_{c}$$

As a general module, SE Block can be integrated into existing CNNs to add an attention mechanism to the network by inferring attention maps in the channel.

### BCNNs

BCNNs include two CNNs without the fully connected layer. This involves inputting the same image, then outputting two feature maps with the same number of feature channels, and then combining them using bilinear pooling to obtain the image descriptor. The BCNNs model *B* can be defined as a quadruple *B* = (*f*_*A*_,*f*_*B*_,*P*,*C*), where *f*_*A*_ and *f*_*B*_ are two feature extraction functions, *P* is a pooling function and *C* is a classification function. BCNNs achieves bilinear pooling using outer product. As an example, given two feature maps A, B of size (*x*,*y*,*z*), firstly reshape them into (x*y,*z*), then the outer product can be calculated which is equal to (*z*,*z*). The feature maps obtained by convolution carries position information, in which several pixels of the original image are convolved into one pixel at the corresponding position. However, the spatial dimension disappears in the outer product of the feature maps, which are called orderless features. BCNNs combine the features of two feature maps in pairs which ignore the location information, so that orderless features are learned and local features can be modeled in a translation invariant way. BCNNs uses CNNs without the fully connected layer as the backbone network, which can be easily integrated into existing CNNs. In the part of backbone network, the parameters of the pretrained model without the fully connected layer can be loaded as the initial parameters, and the rest parameters of BCNNs are initialized randomly. The network structure is a directed acyclic graph that is suitable for standard backpropagation training and can train end-to-end with only image labels.

### Fast BCNNs

**FIGURE 2:**
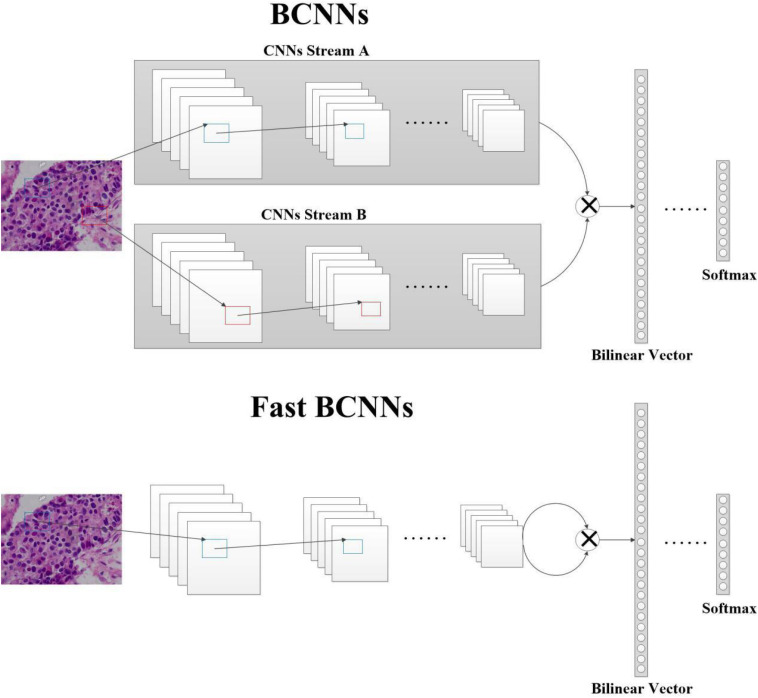
Structure of BCNNs and Fast BCNNs. In BCNNs, a histopathological image is passed through two CNNs, A and B. Their outputs are then sent to the pooling function to get the bilinear vector, then to the classification function to get the prediction result. In Fast BCNNs, a histopathological image is passed through one CNN, and its output is used twice to get the bilinear vector.

We simplify the two CNNs in the BCNNs model into a single CNNs. As shown in Figure 2, the image is input once to get a feature map and bilinear pooling is used to obtain a bilinear vector by the same feature map. The model can be represented as a triple *B*_fast_ = (*f*,*P*,*C*). The feature extraction function receives the image *I* and the location *L*, and outputs a feature vector of size *c*×*D*, which can be expressed as: *f*:*I*×*L*→*R*^*c*×*D*^. For the two images paired in each spatial location, the output of the feature extraction function is combined using outer product:

(5) $$b\,i\,l\,i\,n\,e\,a\,r_{\text{fast}}\,\left(l,i,f\right)=f\,{\left(l,i\right)}^{\text{T}}\,f\,\left(l,i\right)$$

Where *l* ∈ *L*, *i* ∈ *I*. We use sum-pooling to aggregate bilinear features:

(6) $$\phi \,\left(I\right)=\sum_{l\in L}b\,i\,l\,i\,n\,e\,a\,r_{\text{fast}}\,\left(l,I,f\right)$$

The obtained bilinear vector is first computed with the signed square root, then normalized using *L2* normalization, and finally classified using the softmax function.

## Experimental Results

### Performance on Images With Different Resolutions

To elucidate the impact of image resolution on the model, we tested the model’s accuracy using three resolutions, namely 197 × 197 (the minimum input resolution in ResNet50, InceptionV3 and InceptionResNetV2 (Szegedy et al., 2017)), 224 × 224 (the default input resolution for most CNNs) and 448 × 448 (twice the default input resolution for most CNNs) on BreaKHis (Spanhol et al., 2016) dataset. The fine-grained classification models were trained with 8 types of images with 40 × magnification factors. As shown in Table 2, the resolution’s impact on the speed in different models is more significant than its impact on the accuracy. Resolution change from 197 × 197 to 224 × 224 led to the increase of the model’s accuracy by less than 0.4%, while the speed decreased twice. Similarly, the resolution change from 224 × 224 to 448 × 448 led to the increase of the model’s accuracy by less than 1.5% but the speed decreased twice. Higher resolution leads to the increase of the image pixels and requires longer processing. For a fair comparison we used 224 × 224 resolution as this is used in the majority of articles.

**TABLE 2:** Classification accuracies of ResNet50, InceptionV3 and InceptionResNetV2 models on images with different resolution.

	Resolution					
	197 × 197		224 × 224		448 × 448	
Backbone network	Accuracy (%)	FPS	Accuracy (%)	FPS	Accuracy (%)	FPS
Inceptionv3	91.70 ± 0.17	135.4	91.72 ± 0.16	67.7	92.73 ± 0.13	31.3
ResNet50	89.97 ± 0.24	126.1	90.23 ± 0.15	63.3	91.48 ± 0.12	29.0
InceptionResNetV2	91.09 ± 0.23	101.6	91.48 ± 0.12	50.6	92.99 ± 0.15	22.6

*Frames per second (FPS) indicate the speed. Average accuracies ± standard deviations were calculated from 5-fold cross validation of the BreaKHis dataset.*

### Comparison of Different Backbone Networks and Fine-Grained Methods

We compared fine-grained classification performance of three CNNs showing excellent performance in macroscopic image classification, namely ResNet50, InceptionV3 and InceptionResNetV2 using 8 BreaKHis dataset image types with mixed and four individual magnification factors. As weakly supervised fine-grained classification methods do not require annotations, fine-grained feature-based methods and visual attention-based methods can learn more effective features from subtly differentiated image regions (Lin et al., 2015; Jianlong et al., 2017). General methods, such as fine-grained feature-based method BCNNs and visual attention-based method SE Block can be seamlessly integrated into other CNNs. To explore their impact on backbone networks, we integrated them into ResNet50, InceptionV3, and InceptionResNetV2 and compared their performance on BreaKHis dataset with mixed and four individual magnification factors. As shown in the Table 3, the integration of BCNNs and SE Block significantly improved classification. Notably, BCNNs improved classification results more than SE Block. InceptionV3+BCNN model performed best in images classification with mixed magnification factors, suggesting its general applicability.

**TABLE 3:** Performance of ResNet50, InceptionV3 and InceptionResNetV2 models with or without fine grained methods on images with different magnification factors.

		Accuracy (%)				
		Magnification factor				
Backbone network	Fine-grained method	40×	100×	200×	400×	Mixed
Inceptionv3	Not used	91.72 ± 0.16	91.84 ± 0.22	89.83 ± 0.17	87.64 ± 0.22	92.24 ± 0.13
	SE Block	94.99 ± 0.31	94.48 ± 0.24	94.79 ± 0.26	94.23 ± 0.39	94.90 ± 0.23
	BCNNs	95.74 ± 0.21	94.72 ± 0.18	94.78 ± 0.26	94.51 ± 0.23	96.14 ± 0.16
ResNet50	Not used	90.23 ± 0.15	90.17 ± 0.32	89.83 ± 0.54	89.01 ± 0.48	92.48 ± 0.29
	SE Block	92.98 ± 0.32	90.93 ± 0.26	88.09 ± 0.38	92.31 ± 0.28	92.79 ± 0.22
	BCNNs	95.74 ± 0.24	95.44 ± 0.32	94.53 ± 0.25	94.88 ± 0.34	95.27 ± 0.26
InceptionResNetV2	Not used	91.48 ± 0.12	91.52 ± 0.19	92.30 ± 0.26	90.65 ± 0.26	92.62 ± 0.17
	SE Block	94.99 ± 0.28	93.93 ± 0.34	92.31 ± 0.29	91.97 ± 0.36	94.02 ± 0.30
	BCNNs	95.24 ± 0.13	94.16 ± 0.22	94.03 ± 0.29	93.70 ± 0.23	95.08 ± 0.14

*Average accuracies ± standard deviations were calculated from 5-fold cross validation of the BreaKHis dataset.*

To investigate the ability of the model to distinguish between different breast pathology images, we deployed a 2D t-SNE (Van Der Maaten and Hinton, 2008) plot to show the clusters’ performance. The t-SNE is the feature representation of input data passing through the network layers. t-SNE receives the output of the last fully connected layer as input and reduces the high-dimensional data to a 2D representation while maintaining local structures.

**FIGURE 3:**
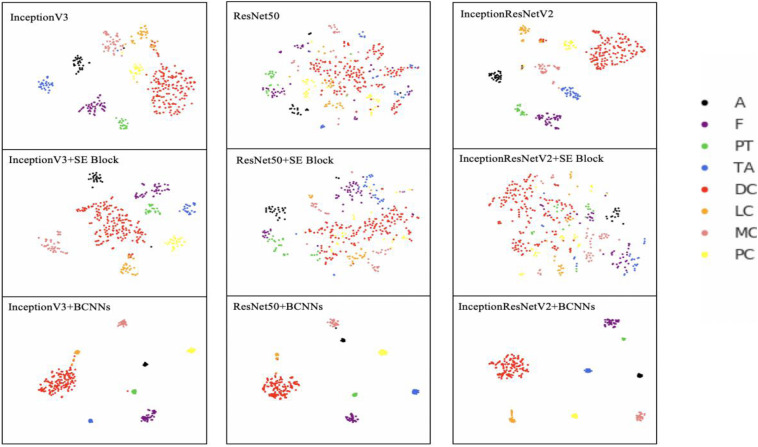
2D t-SNE plot of different networks. InceptionV3, ResNet50, InceptionResNetV2, InceptionV3+SE Block, ResNet50+SE Block, InceptionResNetV2+SE Block, InceptionV3+BCNNs, ResNet50+BCNNs, and InceptionResNetV2+BCNNs models are trained with images with 40× magnification factor. Colored dots represent different breast cancer types. The t-SNE plots with BCNNs are more compact and visible than networks without integrated fine-grained feature-based methods. A (Adenosis); F (Fibroadenoma); PT (Phyllodes Tumor); TA (Tubular Adenoma); DC (Ductal Carcinoma); LC (Lobular Carcinoma); MC (Mucinous Carcinoma) and PC (Papillary Carcinoma).

As shown in Figure 3, the pathological cluster centers of different types are separated from each other in the t-SNE plots with backbone networks. However, in the whole cluster, different pathology clusters have no obvious boundaries and same pathology samples are not compact, especially in the ResNet50 model. It shows that after integrating BCNNs, not only the cluster centers, but also the whole clusters, are far apart. This allows greater distinguishability between clusters.

**FIGURE 4:**
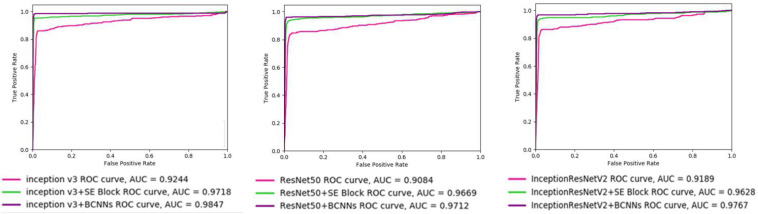
ROC curves of different networks. InceptionV3, ResNet50, InceptionResNetV2, InceptionV3+SE Block, ResNet50+SE Block, InceptionResNetV2+SE Block, InceptionV3+BCNNs, ResNet50+BCNNs, and InceptionResNetV2+BCNNs models are trained with images with 40× magnification factor. The AUC value of models with SE Block and BCNNs are higher than those of the networks without integrated fine-grained feature-based methods.

To investigate the different models’ accuracies, receiver operating characteristic (ROC) curves were drawn. ROC curve is determined by True and False Positive Rates, which are not affected by the sample imbalance in the BreaKHis dataset. Area under the curve (AUC) value of ROC curve reflects the quality of the ROC curve. As shown in Figure 4, the integration of BCNNs or SE Block led to improvements in the AUC value for three backbone networks. Notably, integration of BCNNs showed better results in AUC than SE Block.

**FIGURE 5:**
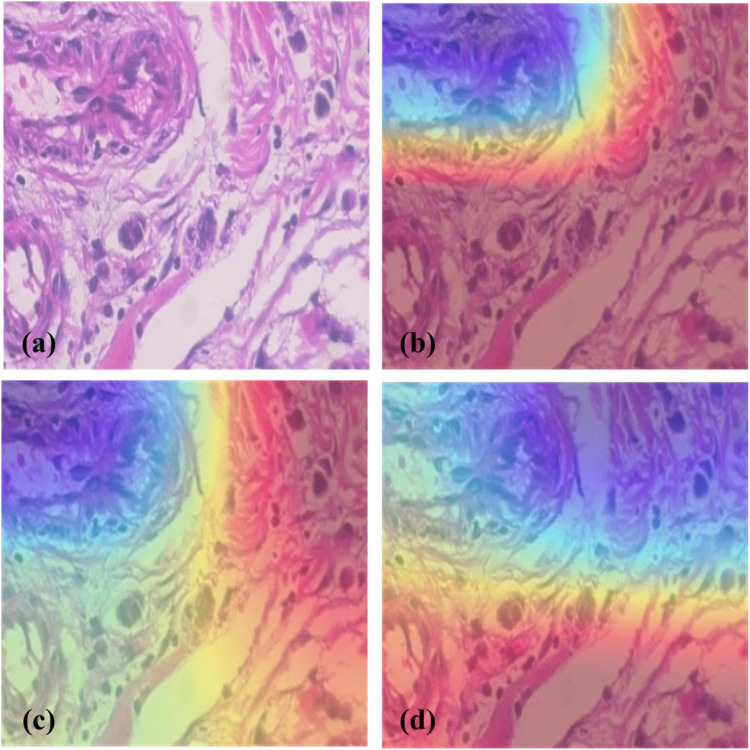
Heatmap generated by different models. **(a)** A slide of Ductal Carcinoma seen in 200× magnification factor, **(b)** Heatmap of InceptionV3, **(c)** Heatmap of InceptionV3+SE Block, **(d)** Heatmap of InceptionV3+BCNNs. The blue, yellow, and red regions represent the degree of positive influence on classification, with the blue regions having the most and the red having the least positive influence. It shows that compared with InceptionV3; the other two methods can detect a larger pathological region.

To analyze which image regions our model focused on, heatmaps of InceptionV3 with fine-grained feature-based methods were plotted (Figure 5). Selvaraju et al. (2017) described visual interpretation of CNNs based on targets’ gradients, called Grad-CAM. Given a specific category and layer, Grad-CAM can perform weighted summation on the feature maps in the convolutional network to obtain the channel weights of the layer and to produce a localization map highlighting important image regions. In the decision making process, Grad-CAM exploits the gradient information flowing into the last convolutional layer of the CNN to assess the importance of features.

Figure 5 shows the heatmaps of different models by Grad-CAM which highlights the importance of regions for classification and demonstrates better focus of the fine-grained methods on pathological regions than ordinary CNNs.

### Comparison of BCNNs and Fast BCNNs

**FIGURE 6:**
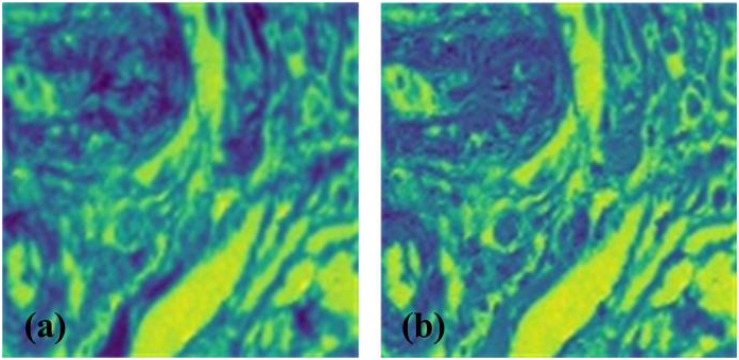
Convolution visualization of InceptionV3+BCNNs. **(a)** Convolution visualization of stream A, **(b)** Convolution visualization of stream B. Similar features were extracted in both streams A and B of CNNs. The darker color represents the activation value. The activation value of the convolution layer aids understanding of the convolution operation. The image activation value after passing the networks can be mapped back to the input pixel space, indicating what input mode leads to a given activation value in the feature map.

Lin et al. (2015) attributed BCNNs’ improvement of fine-grained classification tasks to two-stream CNNs. First stream is used to extract shape features and the second to extract location information. To prove our conjecture, we employed convolution visualization (Zeiler and Fergus, 2014) for BCNNs’ two-stream CNNs (Figure 6). The features used in this experiment were extracted from the feature map obtained from the first convolution layer. The convolution layer in the same position of two-stream CNNs has almost the same response to the image, thus the functions of the two streams in two-stream CNNs are the same (Figure 6).

Ability to extract shape features and location information, respectively, in two-stream CNNs is not the particularity of BCNNs, therefore we simplified it to a single CNN. This new model is termed Fast BCNNs due to its improved speed. We investigated its fine-grained classification performance on 8 types of images of BreaKHis dataset with mixed and four individual magnification factors (Table 4).

**TABLE 4:** Comparison of BCNNs and Fast BCNNs.

		Accuracy (%)						
		Magnification Factor						
Backbone network	Type of BCNNs	40×	100×	200×	400×	Mixed	FPS	NOP (million)
Inceptionv3	BCNNs	95.74 ± 0.21	94.72 ± 0.18	94.78 ± 0.26	94.51 ± 0.23	96.14 ± 0.16	45.5	81.4
	Fast BCNNs	95.99 ± 0.17	95.84 ± 0.16	94.70 ± 0.28	94.51 ± 0.15	95.95 ± 0.19	62.5	59.6
ResNet50	BCNNs	95.74 ± 0.24	95.44 ± 0.32	94.53 ± 0.25	94.88 ± 0.34	95.27 ± 0.26	38.5	84.9
	Fast BCNNs	95.49 ± 0.27	95.78 ± 0.29	94.29 ± 0.26	94.43 ± 0.19	94.96 ± 0.23	58.8	61.3
InceptionResNetV2	BCNNs	95.24 ± 0.13	94.16 ± 0.22	94.03 ± 0.29	93.70 ± 0.23	95.08 ± 0.14	27.8	130.0
	Fast BCNNs	95.99 ± 0.21	93.53 ± 0.18	94.79 ± 0.24	93.78 ± 0.15	95.20 ± 0.17	45.5	75.6

*Frames per second (FPS) and Number of Parameters (NOP) were calculated to evaluate the speed. Average accuracies ± standard deviations were calculated from 5-fold cross validation of the BreaKHis dataset.*

As shown in Table 4 we achieved approximately the same accuracy as the original network structure. However, we improved greatly the speed and reduced the number of parameters. Our method can be used even if pathological features are small and widely distributed, and the resolution reduction of histopathological image results in degeneration. The advantage of BCNNs is not because the two-stream CNNs extract different features, but because the unique bilinear pooling function produces a large number of new orderless features. This benefits fine-grained classification. At the same time, our method deals with the uncertainty of the disease location.

### Experiments in Other Tasks

Most of the previously published breast cancer classification methods based on BreaKHis (Cascianelli et al., 2017; Gupta and Bhavsar, 2017, 2018; Han et al., 2017; Song et al., 2017; Wei et al., 2017; Bardou et al., 2018; Benhammou et al., 2018; Gandomkar et al., 2018; Karthiga and Narasimhan, 2018; Li et al., 2018; Zhang et al., 2018) use binary classification and not fine-grained classification. Furthermore, most binary classification and all fine-grained classification approaches are magnification-specific. Most of these methods are based directly on ordinary or modified CNNs, such as ResNet (Gupta and Bhavsar, 2017; Wei et al., 2017), VGG (Cascianelli et al., 2017; Song et al., 2017), and GoogleNet (Gupta and Bhavsar, 2018). We employed our method (InceptionV3 + Fast BCNNs) to carry out four groups of experiments, including magnification-specific binary classification (MSBC), magnification-independent binary classification (MIBC), magnification-specific fine-grained classification (MSFC), and magnification-independent fine-grained classification (MIFC) (Table 5).

**TABLE 5:** Comparison of our method (InceptionV3 + Fast BCNNs) and other state-of-the-art methods on BreaKHis dataset.

				Accuracy (%)			
				Magnification Factor			
Method	Input	Resolution	Experiment	40×	100×	200×	400×
Cascianelli et al., 2017	Image	224 × 224	MSBC	87.00	85.20	85.00	81.30
Benhammou et al., 2018	Image	Unspecified	MSBC	86.50	83.20	85.40	80.30
Song et al., 2017	Image	Unspecified	MSBC	87.70	87.60	86.50	83.90
Gupta and Bhavsar, 2018	Image	224 × 224	MSBC	94.71	95.90	96.76	89.11
Cascianelli et al., 2017	Image	256 × 256	MSBC	95.80	96.90	96.70	94.90
Wei et al., 2017	Image	224 × 224	MSBC	97.02	97.23	97.89	97.50
Gandomkar et al., 2018	Patch	224 × 224	MSBC	98.52	97.90	98.33	97.66
Our method	Image	224 × 224	MSBC	99.33	99.04	98.84	98.53
Zhang et al., 2018	Image	224 × 224	MIBC	81.20			
Gupta and Bhavsar, 2017	Image	Unspecified	MIBC	88.09			
Karthiga and Narasimhan, 2018	Image	Unspecified	MIBC	93.30			
Our method	Image	224 × 224	MIBC	99.24			
Bardou et al., 2018	Image	Unspecified	MSFC	88.23	84.64	83.31	83.98
Li et al., 2018	Image	Unspecified	MSFC	94.80	94.03	93.85	90.71
Han et al., 2017	Image	256 × 256	MSFC	92.80	93.90	93.70	92.90
Gandomkar et al., 2018	Patch	224 × 224	MSFC	95.60	94.89	95.70	94.63
Our method	Image	224 × 224	MSFC	95.99	95.84	94.70	94.51
Our method	Image	224 × 224	MIFC	95.95			

As shown in Table 5, our method achieves better performance in all four experiments. Our method performs better than the patch-based method (Gandomkar et al., 2018) used in MSBC experiment, and shows comparable performance in MSFC experiment. In addition, our method is not dependent on the magnification factor, thus proving that our model learns a strong scale invariant. Previous methods led to up to 7.65% (Gupta and Bhavsar, 2018) accuracy difference in images with different magnification factors. Furthermore, previous magnification-independent methods have shown worse performance than magnification-specific approaches. Our method shows high accuracy with difference less than 1.5% in experiments with different magnification factors and shows almost no accuracy loss in magnification-independent experiments. Furthermore, we achieved 95.95% accuracy in MIFC experiment.

### Experiments on the In-House Dataset

**FIGURE 7:**
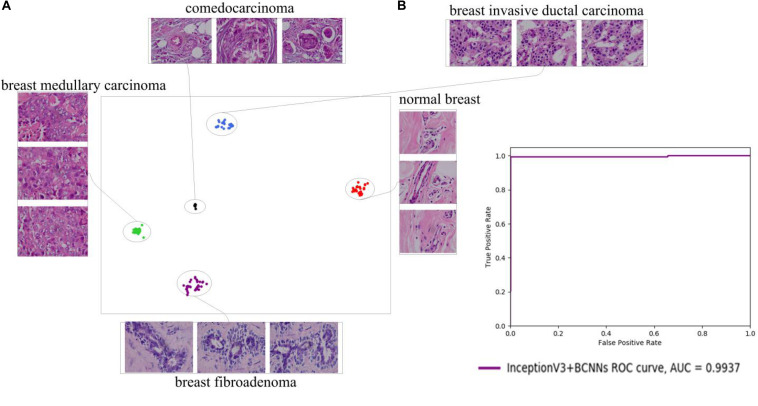
2D t-SNE plot **(A)** and ROC curve **(B)** of InceptionV3+BCNNs on the in-house dataset. The InceptionV3+BCNNs model distinguishes between different types of breast histopathological images, and obtains a high AUC value of 0.9937.

To further evaluate our fine-grained deep learning method, we tested it on an in-house dataset. The tested dataset of histopathological images consisted of five types of breast cancers acquired by a bright field light microscope (Olympus IX53) with 100× oil immersion objectives. In total, 678 histopathological images were collected, including 135 comedocarcinoma, 134 breast fibroadenoma, 134 breast medullary carcinoma, 135 breast invasive ductal carcinoma, and 140 normal breast tissue images. InceptionV3 + Fast BCNNs achieved 99.27% accuracy in all cancer types, which indicates the general applicability of our method (Figure 7).

## Discussion and Conclusion

Most of the currently employed classification models of pathological images are developed for binary classification. Furthermore, a number of similar diseases may occur in the same part of the human body, thus making identification of a specific disease difficult even for trained professionals. While in macroscopic images the target is localized in a certain region, pathological images often contain small and widely distributed pathological features. This important feature of pathological images has been given little attention previously, and consequently they are usually classified with ordinary CNNs. Histopathological images often have a large resolution and thus cannot be directly input into CNNs. To avoid image degradation and loss of classification accuracy caused by image reduction, they are usually first scanned as small patches one by one and predicted separately. Then the patch-level labels are fused to get image-level labels. This is time-consuming, and the model’s performance is affected by the choice of the size of the sliding window and patch. In addition, it is difficult to build association between the patch-level label and the image-level label.

To address these challenges, we proposed a new deep learning approach for fine-grained breast cancer classification. Unlike the previous methods, our approach downsamples histopathological image and directly predicts it, thus greatly speeding up the process and eliminating the indeterminate association between patch and image. This fine-grained classification method captures better the small inter-class and large intra-class variances than patch-based methods. Fine-grained classification algorithms are more sensitive to subtly differentiated pathological regions than ordinary CNNs. To cope with the uncertainty of lesion location, we used bilinear pooling to obtain a large number of orderless features to allow the model to learn a strong translation invariant. Prior knowledge of the selected features is not required in BCNNs, avoiding hand-picking bias. By using BCNNs for orderless features extraction, this approach is able to locate pathological regions. Contrary to binary classification, it provides a more detailed information, thus assisting doctors in the decision making.

Additionally, we improved the accuracy and speed of BCNNs. In the BreaKHis experiments, our method showed excellent performance and is applicable to both binary and multi-classes classification of histopathological images with different magnification factors. Moreover, testing with different magnification shows the robustness of our approach for diverse histopathological images without necessity to train different models separately. Thus our model is applicable to images captured by different devices and can train images across magnification factors. This is valuable particularly for histopathological image analysis where magnifications are not standardized.

Although our method is robust for fine-grained breast cancer classification, some limitations remain. Our method imports pretrained models trained in ImageNet dataset to speed up the training convergence. However, the ImageNet is a macro object dataset, which has fewer common features with microscopic object images. Thus, a more correlated dataset should be considered in pretrain stage. Moreover, besides the fine-grained classification of disease categories, the fine-grained classification of disease stages would be of a high clinical value. This information is not easy to obtain as the existing datasets lack these cases, therefore it will be the focus of the future investigation.

## Data Availability Statement

All datasets presented in this study are included in the article.

## Author Contributions

WL and YZ designed the study. WL wrote the code for the algorithms and drafted the manuscript. MJ and YZ finalized the writing. All authors contributed to the article and approved the submitted version.

## Conflict of Interest

The authors declare that the research was conducted in the absence of any commercial or financial relationships that could be construed as a potential conflict of interest.
